# Experimental Study on Mechanical Properties of Thermally Conductive High-Strength Concrete

**DOI:** 10.3390/ma18030642

**Published:** 2025-01-31

**Authors:** Xiaojun Li, Shenglei Jia, Longgang Chen, Rongjian Shen, Yang Liu, Ruifeng Mou

**Affiliations:** 1 College of Safety Science and Engineering, Xi’an University of Science and Technology, Xi’an 710054, China; mrf13501475134@163.com; 2 College of Geology and Environment, Xi’an University of Science and Technology, Xi’an 710054, China; jiashengleixust@163.com; 3 Shaanxi Transportation Holding Group Ltd., Xi’an 710003, China; lgchensth@163.com; 4 Shaanxi Anjian Investment and Construction Co., Ltd., Xi’an 710003, China; 18391867275@163.com (R.S.); yliusaic@163.com (Y.L.)

**Keywords:** ultra-high-performance concrete, mechanical properties, thermal conductivity, high temperature performance

## Abstract

Ultra-high-performance concrete (UHPC) is considered one of the future building materials due to its excellent performance. UHPC with good thermal conductivity has potential high-value applications in large-scale bridges and nuclear facilities. As a by-product of the coal gasification process, coal gasification slag (CGS) can replace sand in traditional UHPC. In this paper, based on the preparation of UHPC by CGS, silicon carbide (SiC) was added to improve the thermal conductivity of specimens. The application of CGS and SiC as alternatives to quartz sand with varying mix ratios in UHPC was studied. The impact of the substitution ratios of CGS and SiC on fluidity, mechanical properties, and thermal performance was analyzed. The compressive strength and splitting tensile strength of five different kinds of specimens were tested at 7 d, 14 d, and 28 d. The compressive strength and mass loss rate of specimens with five different ratios were also determined under five different temperature conditions (110 °C, 200 °C, 300 °C, 400 °C, and 500 °C). The results show that the maximum compressive strength of 28 d can reach 159.5 MPa and the splitting strength is 15.30 MPa. The addition of SiC can improve the thermal conductivity and thermal stability of concrete. The compressive strength of all specimens is improved after high-temperature treatment. When substitution rate of SiC reaches 100%, the compressive strength of the specimens is up to 182.2 MPa. With the increase in temperature, the concrete burst phenomenon occurs above 300 °C. It is observed that the high-temperature burst resistance of the specimens with low strength is better than that of the specimens with high strength. Two specimens were scanned with Industrial Computerized Tomography (ICT) and the microstructures of the specimens were compared. It was found that the samples with higher SiC substitution rates had more minor total pore defects and larger pores.

## 1. Introduction

In recent years, UHPC has been widely used in infrastructures. However, the current research on the thermal performance of UHPC is not enough. Revealing the spalling mechanism and clarifying the mechanical properties of UHPC at high temperature, as well as improving the high-temperature resistance of UHPC, are the key issues to promote the application of UHPC in practice.

Researchers have conducted experiments and studies on thermal performance and fire resistance of UHPC. Liang et al. [1] developed a UHPC mixture with excellent fire-resistance properties, which retained 69% of its original compressive strength after being exposed to 1000 °C. Bolina et al. [2] observed that when UHPC is exposed to high temperatures, its thermal expansion and strength reduction are less than those of normal concrete, but its thermal conductivity and mass loss are greater. Chen [3] showed that the damage of concrete under high temperature is a result of a combination of factors, and the relationship between them is very complex. Du et al. [4] observed that the risk of spalling in high-strength concrete (HSC) primarily relies on factors such as the proportions of the mix, the rate of heating, the moisture content in the specimens, the density and shape of the samples, the type of aggregate used, and the configuration of reinforcements. Banerji and Kodur [5] determined that the compressive strength of UHPC decreases more rapidly than that of traditional concrete when subjected to elevated temperatures, primarily because of spalling and the internal stress generated by water pressure within the pores. Ruiyu et al. [6] observed that ordinary concrete follows the following patterns as the temperature changes: temperature < 100 °C—the free water inside the concrete gradually evaporates, forming many capillary cracks as well as pores inside the concrete; temperature between 200 °C and 300 °C—the free water inside the concrete has all evaporated and the bonding water in the cement gel water starts to come off. Liang et al. [7] found that the UHPC tested in a hot state of 200 °C suffered severe degradation in strength and rigidity due to great internal vapor pressure. Much less such degradation happened to the UHPC under 400 °C. Hernández-Figueirido et al. [8] acknowledged that there are only a limited number of studies examining the mechanical properties of UHPC when subjected to high temperatures. Furthermore, the discrepancies in the findings are largely influenced by factors such as the procedures used in testing, the dimensions of the specimens, the rates of heating, and the proportions of the concrete mixtures employed. Qin et al. [9] concluded that reducing the moisture content is an effective way to improve spalling resistance. The dense microstructure of UHPC is related to the high risk of explosive spalling under fire conditions. Adding PP fibers (PPFS) is considered to be an effective measure to improve the explosion problem of UHPC in a high-temperature environment. Yang et al. [10] noted that the compressive strength of UHPC made with PPFS and higher amounts of steel fibers diminishes when exposed to temperatures ranging from 300 °C to 500 °C. Zhou et al. [11] concluded that UHPC with quartz sand does not exhibit good fire-resistance properties and is also highly susceptible to fire-induced spalling. Zheng et al. [12] found that the thermal conductivity of reactive powder concrete was greater than that of calcium aggregate at room temperature due to the higher crystallinity of silicon aggregate.

Coal is the largest fossil strategic energy reserve in China; there were 4.56 billion tons of coal production during 2022 [13]. Driven by carbon neutral and carbon emission reduction policies, the clean utilization technology of coal has also achieved a continuous breakthrough [14]. Coal gasification represents one of the most environmental friendly methods for indirect liquefaction of coal, with CGS being the by-product of the liquefaction process that constitutes 15–20% of the raw materials [15]. However, the industrial process brings benefits; the main treatment methods for CGS are landfill and stacking, which will result in environmental risks and economic pressure. Therefore, it is essential to use CGS in large-scale industrial process.

CGS is divided into coal gasification fine slag (CGFS) and coal gasification coarse slag (CGCS) due to the different discharge positions of the production process. Nevertheless, only a limited number of studies have explored the use of CGCS as a substitution for fine aggregate in UHPC. Therefore, CGS exhibits significant potential for use as a fine aggregate substitution in UHPC. The largest particle size of CGCS ranges from 2 to 5 mm, comparable to the particle size of river sand utilized in UHPC; therefore, it can be use without mining and grinding [16]. Lian et al. [17] found that CGCS has potential for application in UHPC as an RS substitution. The mechanical properties of specimens with CGCS substitution ratios of <25% satisfy the strength requirement of non-structural UHPC. Li et al. [18] investigated the reaction mechanism of CGS in relation to cement; in contrast to CGFS, the presence of abundant active mineral phases in CGCS promoted the cementitious reaction and improved the strength of the mortar. Yoshitaka [19] found that the use of fine aggregates for concrete is adequate and possible, and the experiments indicated that the compressive strength and durability of such slag concrete did not show differences from those of concrete made with sand. The mineral components of CGS are similar to fly ash, but also have the corresponding physical and chemical characteristics. In addition to carbon, the chemical composition of CGS also includes SiO_2_, Al_2_O_3_, and other components, which have potential pozzolanic activity [20]. According to the research of Luo et al. [21], the content of calcium hydroxide in the cement hydration product is greatly reduced when fine ash and CGS are added. On the whole, the synergistic use of fine ash and CGS may have a better effect on the fluidity and strength of cement-based materials. The carbon content in fine slag is higher. Based on research conducted by Blaisi et al. [22], milling of high-temperature arc gasificaion slag to a finer particle size increased heat evolution at all replacement percentages, and resulted in improvements in compressive strength at 28 days of age. Jiao et al. [23] observed that CGS with a higher crushing value, as the substitution material of quartz sand and river sand in UHPC under normal curing conditions, could improve the compressive strengths of concretes. When the substitution degree was 75%, the compressive strength reached the maximum. Wei et al. [24] found that CGS can be used as a raw material to prepare nonburnt bricks; at higher CGS dosages, the prepared nonburnt bricks could meet the MU20 standard [25]. Zhang et al. [26] found that because CGS consists of a pozzolanic reactive mineral, in an alkalinity situation, the CGS mortar strength can be improved; it can be applied to road engineering material to replace the fine aggregate and partial cement.

This study investigated the application of SiC and CGS in UHPC. Quartz sand was replaced with SiC and CGS to improve the thermal conductivity and low-cost efficiency. The fluidity, mechanical properties, and thermal performance with different mix ratios were examined. The findings of this paper aim to provide insights for enhancing and revealing the performance of UHPC under high-temperature conditions, while also providing a foundation for the advancement of innovative and environmentally friendly industrial technologies utilizing CGS, which is essential for the low-carbon coal chemical engineering and construction industry in China.

## 2. Materials and Methods

### 2.1. Raw Materials

#### 2.1.1. Binders

In the present investigation, the supplementary cementitious material used to produce UHPC included fly ash (FA), silica fume (SF), limestone flour (LF), and ordinary Portland Cement P.O 52.5. The chemical composition of the binders is listed in Table 1. To this mixture, a poly-carboxylic ether super plasticizer (SP) with 55% solid content was also added.

#### 2.1.2. Fine Aggregates

SiC and CGS were used as fine aggregates. The chemical composition of SiC chosen is shown in Table 2. The CGS used in the test was purchased from a coal chemical company in Shenmu Shaanxi. The chemical composition of SiC chosen is shown in Table 3. According to the statistics of preliminary screening, it is known that the particles of the batch of CGS with a particle size less than 2 mm accounted for about 60%, the particles with a size of 2–5 mm accounted for about 30%, and the rest were particles with a size of more than 5 mm.

#### 2.1.3. Steel Fibers

Steel fibers (STFs) with lengths between 6 and 13 mm may improve the mechanical properties of UHPC. As a result, 10 mm long straight steel fibers with a 0.2 mm diameter were employed to produce UHPC. The tensile strength and density of STF were 3000 MPa and 7850 kg/m^3^.

### 2.2. Mix Design

SiC is dense and with poor absorbing property and the water absorption rate of CGS is much higher. In order to control workability, the water–binder ratio was initially set at 0.22 and the fluidity was set from 180 to 200 mm in the first experimental design stage. Table 4 shows the detailed composition of 16 designed mixtures and fluidity test results. Through analysis of the fluidity test results, the best ratio was determined in final design stage, as shown in Table 5.

### 2.3. Specimen Preparation

Mixtures were prepared with procedures of previous studies [26]. Step 1: Aggregates and binders were mixed with low speed for 3 min. Step 2: Mixtures were mixed continuously at a high speed for 7 min, while gradually adding 75% solution of SP and water. Step 3: The left water and SP were incorporated and blended at a low speed as the mixture’s fluidity neared the target consistency. Step 4: STF was gradually added and stirred at 60 rpm until a fresh paste was achieved.

The mixtures were placed into molds measuring 20 mm× 20 mm× 20 mm and then vibrated for 60 s to eliminate bubbles; then molds were covered to prevent moisture loss from the surface of the specimens and kept in an environment at 20 ± 1 °C and relative humidity of 95% for 48 h. Once demolded, the cubic specimens were placed into stream curing at 95 ± 1 °C and relative humidity of 95% for 24 h. Specimens were stored under conditions of 20 ± 1 °C and relative humidity of 95% until they were tested at age of 7 days, 14 days, and 28 days [27].

### 2.4. Test Methods

#### 2.4.1. Fluidity Test

The fluidity of the freshly prepared concrete specimen was tested by measuring the flow diameter of slurry. Fluidity tests are carried out by the C230/C230M-14 standard [28]. Each group was measured three times during the fluidity test and the average of three results was considered as the test result.

#### 2.4.2. Mechanical Test

The splitting and compressive strength of three samples from each group were evaluated on days 7, 14, and 28 with a testing machine, which operated at a loading rate of 0.03 mm/s. The average of three results of each group was considered as the test result for each group. Formulas (1) and (2) were employed to calculate the splitting and compressive strength of specimens, as follows:

(1) $$F_{ts}=\frac{2F_{t,\max}}{\pi A}$$

where *F_ts_* represents the splitting tensile strength of concrete cube specimens; *F_t_*_,max_ is the failure load of specimens; *A* is the bearing area of specimens, which was set as 380 mm^2^ in this study.

(2) $$f_{cc}=\frac{F_{c,\max}}{A}$$

where *f_cc_* represents the compressive tensile strength of concrete cube specimens; *F_c_*_,max_ is the failure load of specimens; A is the bearing area of specimens, which was set as 380 mm^2^ in this study.

#### 2.4.3. Water Absorption Test

The water absorption of the samples was assessed on the 28^th^ day following the ASTM C1585-2013 standard [29]. After conducting mechanical tests, three parallel samples measuring 20 mm × 20 mm × 20 mm were prepared. The average values of the three water absorption results for each group were calculated.

#### 2.4.4. Thermal Performance Test

Thermal conductivity was determined with a Hot Disk (Sweden Hot Disk TPS2500S, Göteborg, Sweden). Samples for thermal conductivity were prepared with a diameter of 4 cm and thickness of 5 mm.

The specific heat capacity of the materials was tested by the indirect DSC method with a standard material, sapphire [30]. The concrete specimens cured to 28 d were ground into powder; 3–5 mg of them was selected as the test sample for specific heat capacity.

#### 2.4.5. High-Temperature Mechanical Properties

The specimens were first preheated and baked in an electric blast drying oven at 110 °C for 6 h to adapt to the high-temperature environment. Then, the specimens were placed in a muffle furnace. The temperature was raised to the target temperature (110 °C, 200 °C, 300 °C, 400 °C, and 500 °C) with a heating rate of 10 °C/min, and was kept at the target temperature for 30 min to simulate the fire environment. After fully cooling, the specimens were taken out. The appearance and quality changes of the specimens before and after high-temperature heating were compared. The compressive strength for each mix ratio and temperature point was determined by averaging three results.

#### 2.4.6. Industrial Computerized Tomography (ICT) Scan

Two cubic specimens were scanned with a scanning layer thickness of 0.2 mm for continuous scanning of 100 layers by a high-precision industrial CT system model AX2000 (Aoyun Electronic Technology Co., Ltd., Xi’an, China). A three-dimensional model was reconstructed with scanning images. The morphology and three-dimensional pore distribution of the samples were analyzed.

## 3. Results and Discussion

### 3.1. Mixture with Different Ratios

#### 3.1.1. Fluidity

Fluidity is used to evaluate the working performance of concrete. Table 4 shows that when the fluidity was 180–200 mm, the w/b increased with the increasing CGS substitution ratios, in the range of 0.19–0.40. The fluidity of UHPC is dominated by particle packing destiny and surface property [31]. In comparison to SiC, CGS features smaller particles and greater specific surface area [32], which enables it to absorb water from the fresh concrete during mixing. Consequently, the use of CGS influenced the fluidity of the slurry.

#### 3.1.2. Mechanical Properties

**Figure 1:**
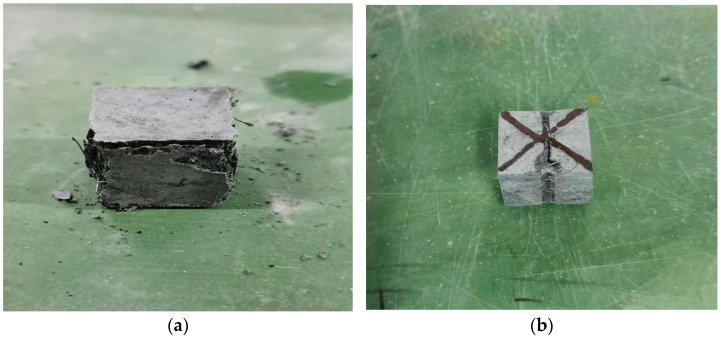
Specimen failure appearance: (**a**) compressive specimen, (**b**) splitting specimen.

**Figure 2:**
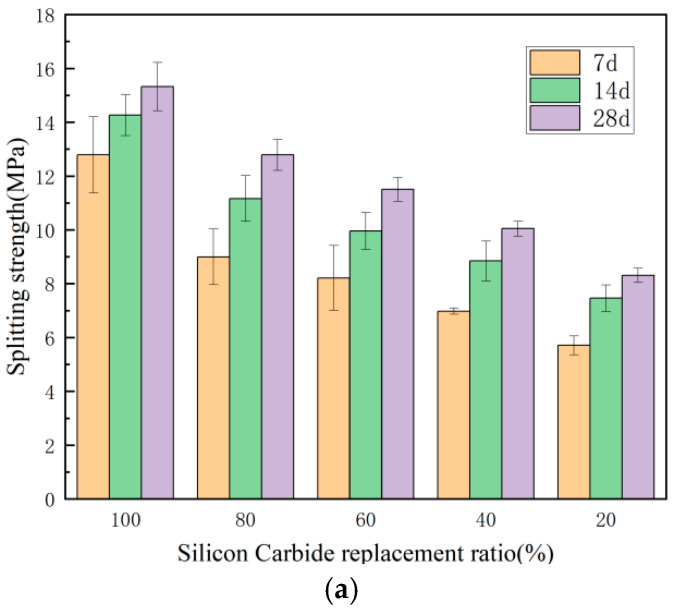

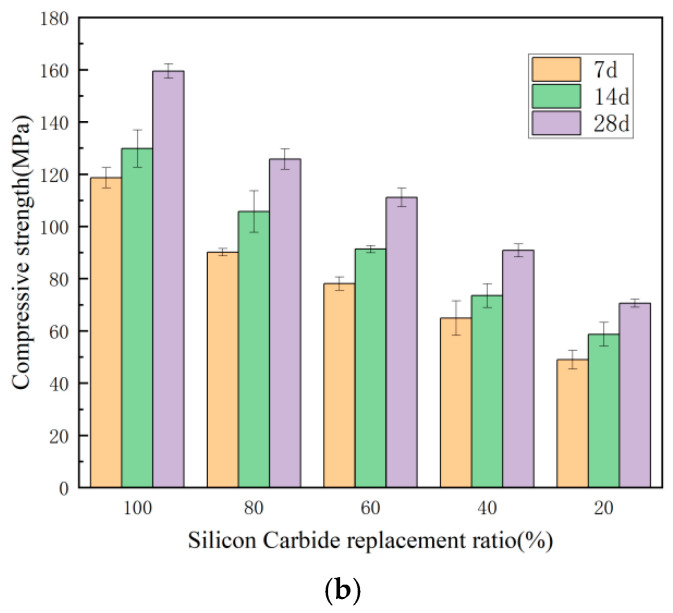
Mechanical strength of specimens with different SiC substitution ratios on days 7, 14, and 28: (**a**) splitting strength and (**b**) compressive strength.

The specimen’s appearance following failure is illustrated in Figure 1. Figure 2 displays the splitting tensile and compressive strengths of the samples containing SiC at various substitution ratios on days 7, 14, and 28.

As depicted in Figure 2, an upward trend in the CGS substitution ratio correlates with a decline in the specimen’s splitting strength. On the 7th day, the splitting strength of specimens with CGS substitution ratios ranging from 0% to 60% varied between 7.0 and 12.8 MPa, while it decreased to 5.7 MPa at an 80% substitution ratio. On the14th day, specimens with CGS substitution ratios of 0–60% exhibited splitting strengths between 8.8 and 14.3 MPa, and dropping to 7.5 MPa at the 80% substitution ratio. On the 28^th^ day, the samples with CGS substitution ratios of 0–60% showed splitting strengths fluctuating from 10.0 to 15.3 MPa, and the value fell to 8.3 MPa with 80% substitution. As the curing period extended, the splitting strengths of the specimens increased alongside the CGS substitution ratio. The materials maintained commendable splitting tensile strength, exhibiting better mechanical properties compared to conventional concrete.

As the ratio of CGS substitution rises, there is a gradual decline in the compressive strength of concrete. On the 7th day, the measurements of the compressive strengths of SC100 through SC20 were 118.6, 90.1, 78.1, 64.9, and 49.0 MPa, respectively. For days 14 and 28, the strengths measured for SC100 to SC20 were 129.8, 105.7, 91.3, 73.5, 58.7 MPa, and 159.5, 125.8, 111.1, 90.8, and 70.6 MPa, respectively. The observed decrease in strength can be attributed to the lower strength of CGS aggregates compared to SiC, along with the detrimental effect of CGS on the hydration process of concrete. This leads to a concrete mix that is characterized by reduced density, increased porosity, and a more porous structure due to the presence of CGS aggregates. Regarding curing age effects, the compressive strength improved from day 7 to day 28. The compressive strength remained high, demonstrating superior mechanical properties compared to conventional concrete.

#### 3.1.3. Water Absorption

**Figure 3:**
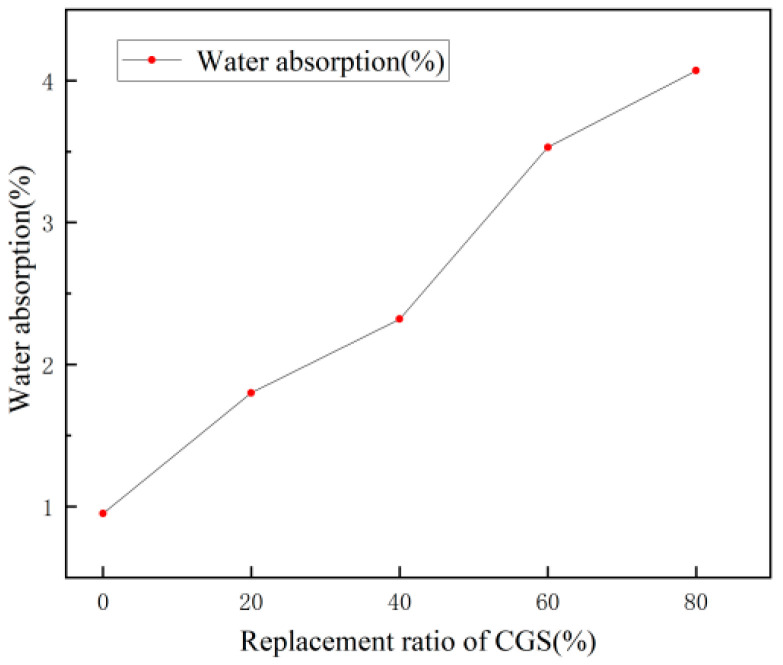
Water absorption of specimens with different CGS substitution ratios.

Figure 3 shows that the water absorption of the concrete increases with the increase in the amount of CGS. The water absorption of SC20 reached 4.07%, which was higher than that of SC100. The water absorption capacity of CGS surpasses that of SiC, whose elevated level of water absorption results in a greater retention of moisture within the concrete specimen, consequently affecting its water content and density.

### 3.2. Measurement of Thermal Performance of Specimen

#### 3.2.1. Thermal Conductivity

**Figure 4:**
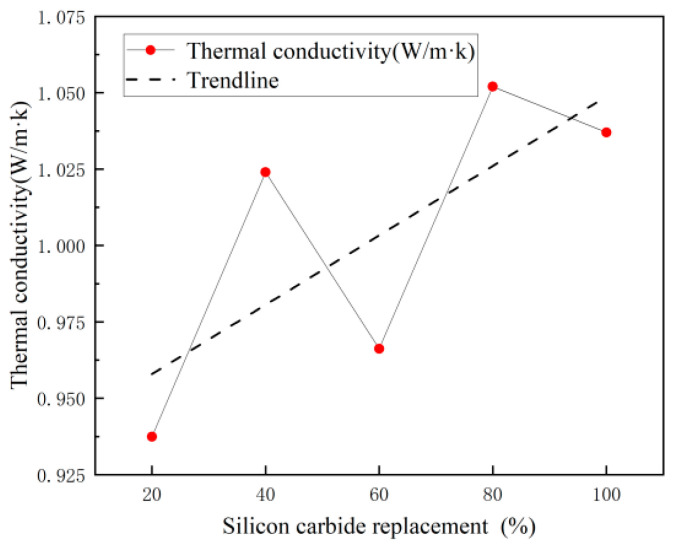
Thermal conductivity of specimens with different SiC substitution ratios.

The thermal conductivity of specimens under different mix ratios is shown in Figure 4. When the substitution rate of SiC is 20%, the thermal conductivity of the specimen is the lowest, which is 0.937. It reached the highest when SiC substitution was 80%, which is 1.052 and about 1.4% higher than that when the substitution rate was 100%. The figure shows that the thermal conductivity increases with the substitution rate of SiC, indicating that the incorporation of SiC can improve the thermal performance of concrete.

#### 3.2.2. Specific Heat Capacity Test

**Figure 5:**
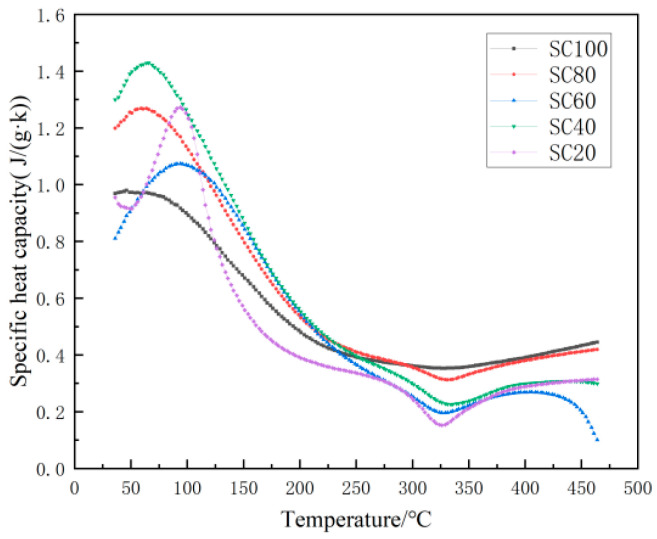
Specific heat capacity of specimens with different SiC substitution ratios at each temperature.

Figure 5 shows the change in the specific heat capacity of specimens with different temperatures under different SiC substitution rates. A peak and valley occurs at the temperatures of 60–100 °C and 300–350 °C, respectively. The peak of 60–100 °C is attributed to water evaporation, which indicates that the specific heat capacity is greatly affected by the water content below 100 °C. The cause of the valley occurring at 300–350 °C is still unclear, and should be further studied.

### 3.3. Fire Resistance Performance Test

#### 3.3.1. High Temperature Heating

**Figure 6:**
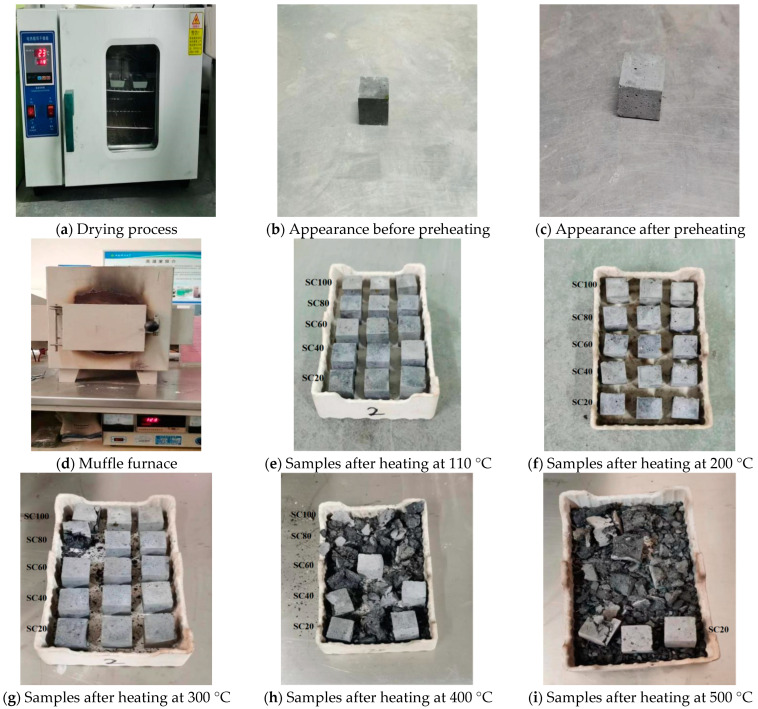
Samples before and after heating.

Figure 6 shows that the all five groups of specimens did not spall at 110 °C and 200 °C, although the appearance of the color became lighter. At 300 °C, it is observed that one specimen of each group of SC100 and SC80 was spalled. At 400 °C, it is observed that three specimens of each group of SC100 and SC80 were spalled, two specimens of group of SC60 were spalled, and one specimen of each group of SC40 and SC20 was spalled. At 500 °C, it is observed that all specimens of each group SC100, SC80, SC60, and SC40 were spalled, while two specimens of group SC20 kept well. We can observe that with increasing the temperature, severe damage occurred in all specimens. Serious damage to all samples happened between temperatures of 300 and 400 °C, which corresponded to the observation of specific heat capacity change at temperatures of 300–350 °C in Figure 5. Inner structure change at temperatures of 300–350 °C is critical to the fire resistance of the designed concrete samples.

#### 3.3.2. Mass Loss Rate

The mass loss rate indicates the stability of concrete and its resistance to stripping under elevated temperatures. Formula (3) was used to assess the splitting strength of the specimen, as detailed below:

(3) $$q=\frac{m_{b}-m_{a}}{m_{b}}\times 100\%$$

where *q* represents the mass loss rate of specimens; *m_b_* is the weight of the specimen before high-temperature treatment; *m_a_* is the weight of the specimen after high-temperature treatment.

The results of mass loss rate after heating at each temperature are shown in Table 6. It is observed the mass loss of specimens with different proportions ranges from 1.90 to 11.17%, and the mass loss value rate of each specimen went up with the increase in temperature. This is probably caused by the water loss or the residual coal burning of CGS with increasing temperature.

#### 3.3.3. Compressive Properties After Heating

**Figure 7:**
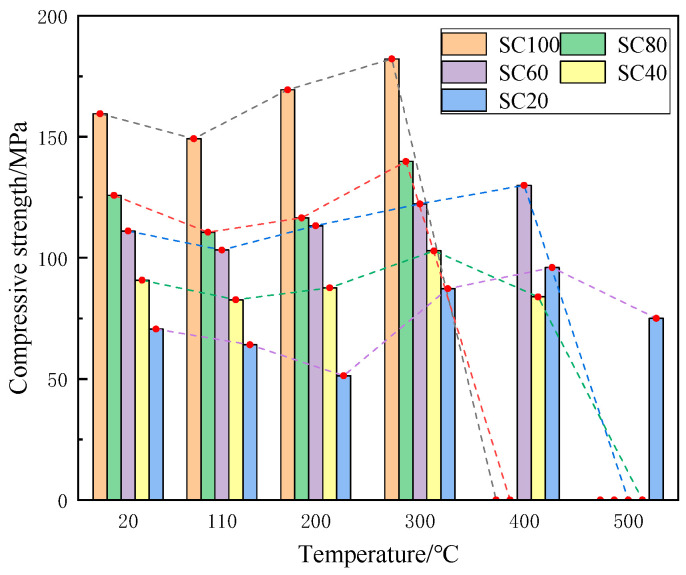
Compressive strength test results after heating at different temperatures.

The compressive strength test results after heating are shown in Figure 7. The compressive strength of the five groups’ specimens all show a trend of decreasing and then increasing with the increase in temperature. Under high temperatures, there are three main stages of concrete strength changes: the initial strength loss stage, the strength recovery stage, and the permanent strength loss stage [33].

The compressive strength of all the specimens decreased from 20 to 110 °C when SiC substitution reached 100% and 80%; the compressive strength gradually increased and reached a peak at 300 °C, then spalled at 400 °C. The peak compressive strength of 60% SiC substitution was 130.0 MPa at 400 °C, and that of 40% SiC substitution was 102.9 MPa at 300 °C. The compressive strength of 20% SiC substitution decreased from 70.6 MPa to 51.3 MPa at 20–200 °C, and the compressive strength gradually increased from 200 °C to 400 °C. The compressive strength reached a peak of 96.0 MPa at 400 °C and decreased to 75.0 MPa at 500 °C.

### 3.4. Microstructure Analysis

**Figure 8:**
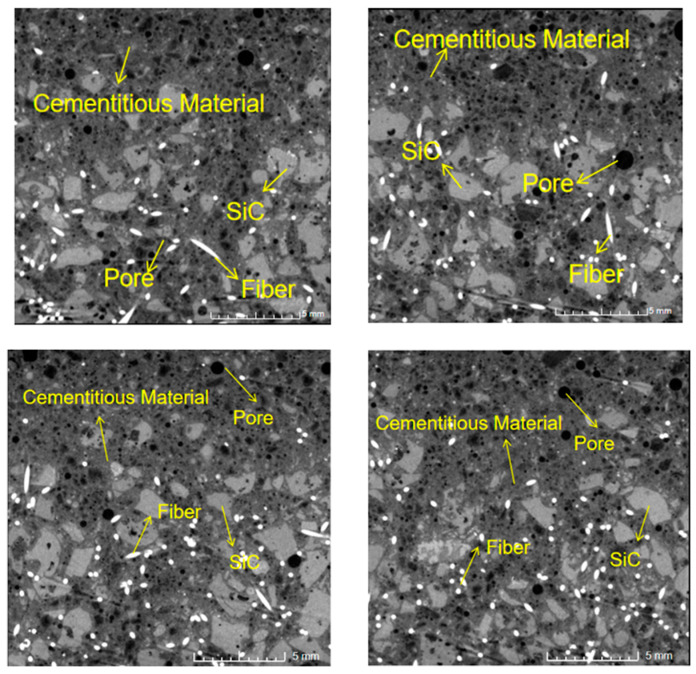
ICT image of SC20.

**Figure 9:**
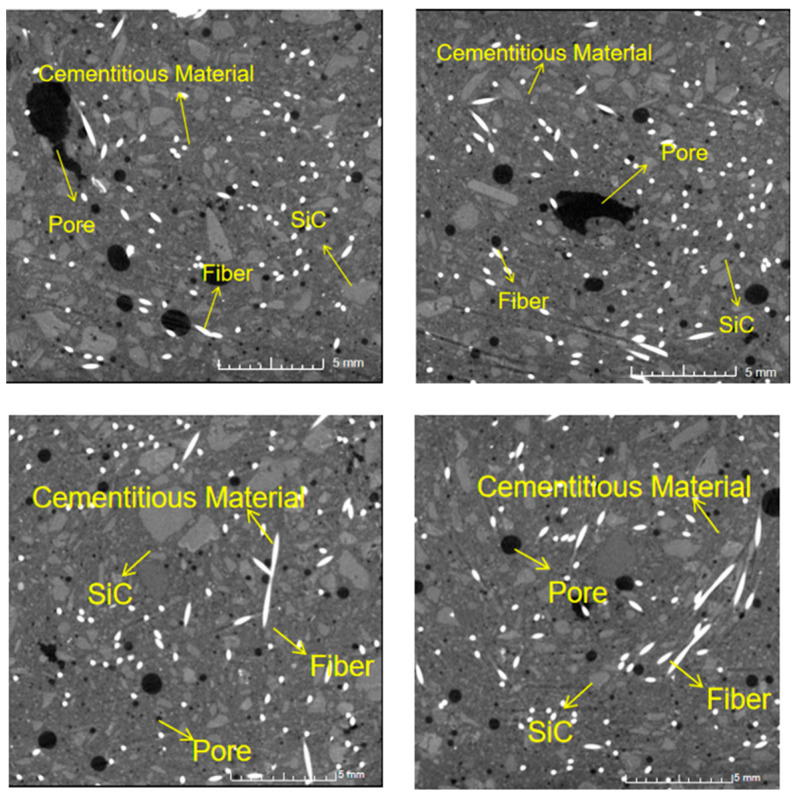
ICT image of SC100.

Figure 8 and Figure 9 are the typical ICT scan images of SC20 and SC100 samples at 28 days without heating. As shown in the ICT images, the brightest pattern is the steel fiber, which was uniformly distributed in the concrete, and the darkest is the pore. It is observed that the materials in SC100 distribute more uniformly than in SC20. The pores of SC20 are not connected and showed a segregation phenomenon, which is attributed to non-uniform vibration of mixing and the internal pores not being fully discharged during the molding process.

**Figure 10:**
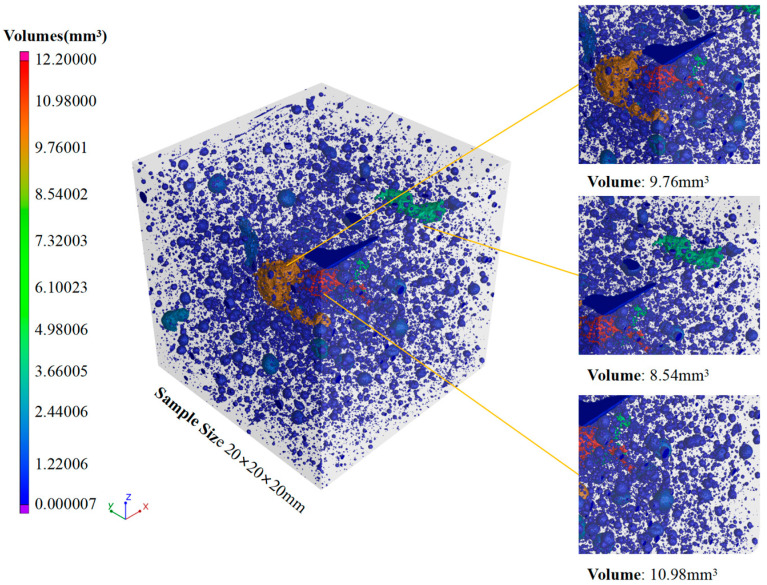
Reconstruction pore map of SC100 sample.

**Figure 11:**
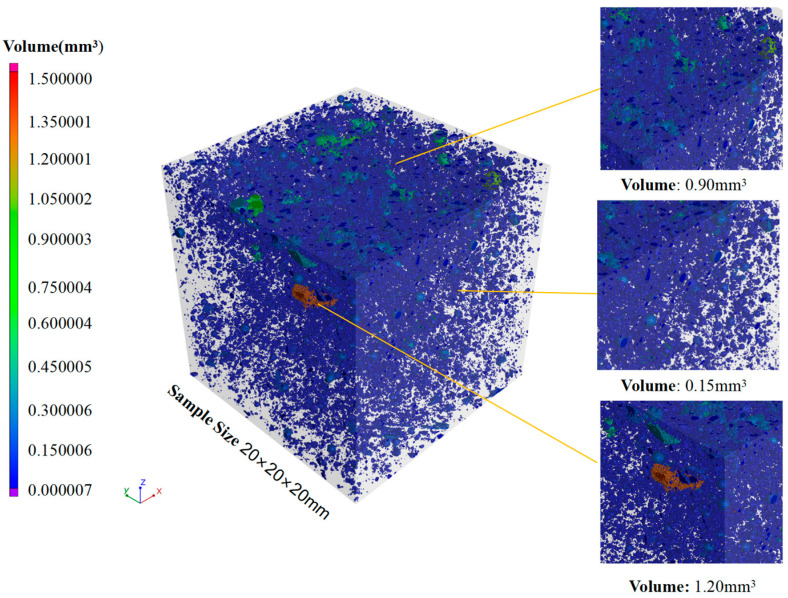
Reconstruction pore map of SC20 sample.

The reconstruction pore maps of SC100 and SC20 are show in Figure 10 and Figure 11. It is observed that the larger pores existed in the SC100 specimens, for which the volume of the largest pore in the specimens was 10.98 mm^3^. The specimen of SC20 has smaller pores; the volume of all the pores is under 1.50 mm^3^, which is much lower than in SC100. The larger pores in the SC100 sample are possibly the reason for the more severe spalling phenomenon.

## 4. Conclusions

Based on the experimental method, this paper reduces the amount of cement by adding fly ash admixture to the cementitious system, replaces the quartz sand in the traditional UHPC with SiC and CGS in the aggregate, and uses solid waste materials based on further improving the thermal conductivity of concrete. The mechanical properties of five different ratios, six different temperatures, and two kinds of strength were tested. The main tests include the fluidity test, cube compressive strength test (7 d, 14 d, 28 d), splitting tensile strength test (7 d, 14 d, 28 d), water absorption, thermal conductivity, specific heat capacity, mass loss rate, and cube compressive strength test after high-temperature treatment, and ICT analysis and comparison of the microstructure of SC100 and SC20 specimens.

1. The mix ratio was optimized with the w/b and fluidity; the fluidity of the five chosen mix ratios was set between 180 and 200 mm, and the fluidity and water absorption increased with the increase in the CGS substitution ratio.
2. CGS and SiC has the potential for application in UHPC as quartz sand. With increasing SiC substitution ratios, the splitting and compressive strength of specimens increased on days 7, 14, and 28. Compared with ordinary concrete, it still shows better compressive and splitting strength.
3. The test results of thermal conductivity and specific heat capacity shows that the addition of SiC can improve the thermal conductivity and the thermal stability of concrete. Based on the testing results of specific heat capacity, the peak and valley occurs at T = 60–100 °C and T = 300–350 °C, which correspond with the high temperature spalling phenomenon of the specimen at 300 °C.
4. With the increase in temperature, the spalling phenomenon is more severe, and the high-temperature resistance of the specimens with lower substitution of SiC is better than that of the specimens with higher substitution. Through ICT scanning, the specimen of SC20 has smaller pores, which are much smaller than those in SC100. Both the substitution of SiC and pore distribution can influence the fire resistance performance of concrete. The inner structure change at temperatures of 300–350 °C is critical to the fire resistance of the designed concrete samples. More research will be continued to explain those experimental phenomena in future study.

## Figures and Tables

**Table 1:** Physical properties of binders (%).

Material	SiO_2_	Al_2_O_3_	Fe_2_O_3_	CaO	MgO	Na_2_O	K_2_O
P.O 52.5	20.96	5.70	3.72	57.81	1.11	0.29	0.17
FA	42.43	21.83	12.81	15.12	2.12	2.04	1.02
SF	95.19	-	0.13	-	0.80	-	-
LF	3.45	1.47	0.24	52.12	0.77	-	-

**Table 2:** Chemical properties of SiC (%).

Material	SiO_2_	SiC	Fe_2_O_3_	F.C	Granularity (D50)/μm
SiC	0.45	98.41	0.17	0.13	10

**Table 3:** Chemical properties of CGS (%).

Material	SiO_2_	Al_2_O_3_	Fe_2_O_3_	CaO	MgO	Na_2_O	K_2_O
CGS	41.19	15.46	17.70	12.61	1.68	1.71	1.36

**Table 4:** Mix ratio and fluidity test results in 1^st^ design stage (kg/m^3^).

Mixture	SiC	CGS	P.O 52.5	FA	SF	LS	STF	SP	Water	W/B	Fluidity (mm)
SC100	1200	0	600	400	200	100	100	50	256	0.24	240
SC100	1200	0	600	400	200	100	100	50	234	0.22	200
SC100	1200	0	600	400	200	100	100	13	228	0.19	170
SC100	1200	0	600	400	200	100	100	13	234	0.19	198
SC80	960	240	600	400	200	100	100	50	249	0.23	169
SC80	960	240	600	400	200	100	100	50	255	0.23	174
SC80	960	240	600	400	200	100	100	50	270	0.25	190
SC60	720	480	600	400	200	100	100	50	276	0.25	173
SC60	720	480	600	400	200	100	100	50	288	0.26	177
SC60	720	480	600	400	200	100	100	50	294	0.26	189
SC40	480	720	600	400	200	100	100	50	390	0.34	173
SC40	480	720	600	400	200	100	100	50	420	0.36	221
SC40	480	720	600	400	200	100	100	50	390	0.34	192
SC20	240	960	600	400	200	100	100	50	420	0.36	170
SC20	240	960	600	400	200	100	100	50	460	0.37	178
SC20	240	960	600	400	200	100	100	50	465	0.40	201

**Table 5:** Mix ratio in final design stage (kg/m^3^).

Mixture	SiC	CGS	P.O 52.5	FA	SF	LS	STF	SP	Water	W/B	Fluidity (mm)
SC100	1200	0	600	400	200	100	100	50	234	0.22	200
SC80	960	240	600	400	200	100	100	50	270	0.25	190
SC60	720	480	600	400	200	100	100	50	294	0.26	189
SC40	480	720	600	400	200	100	100	50	390	0.34	192
SC20	240	960	600	400	200	100	100	50	465	0.40	201

**Table 6:** Mass loss rate after heating at each temperature.

T/°C	Mixture	*m_b_* (g)	*m_a_* (g)	*m_b_* − *m_a_* (g)	Mass Loss Rate (%)
110	SC100	21.03	20.63	0.4	1.90
	SC80	19.49	18.82	0.67	3.44
	SC60	18.93	18.54	0.39	2.06
	SC40	17.27	16.61	0.66	3.82
	SC20	16.19	15.61	0.58	3.58
200	SC100	20.44	19.97	0.47	2.30
	SC80	19.08	18.48	0.6	4.19
	SC60	18.16	17.66	0.5	2.75
	SC40	15.87	15.23	0.64	4.03
	SC20	15.51	14.86	0.65	4.19
300	SC100	20.63	19.18	1.45	7.03
	SC80	18.82	17.59	1.23	6.54
	SC60	18.54	17.06	1.48	7.98
	SC40	16.61	15.34	1.27	7.65
	SC20	15.61	14.47	1.14	7.30
400	SC100	20.50	0	—	—
	SC80	18.82	0	—	—
	SC60	18.32	16.44	1.88	10.26
	SC40	16.08	14.39	1.69	10.51
	SC20	15.56	13.94	1.62	10.41
500	SC100	20.50	0	—	—
	SC80	18.82	0	—	—
	SC60	18.32	0	—	—
	SC40	16.08	0	—	—
	SC20	15.58	13.84	1.74	11.17

## Data Availability

The original contributions presented in this study are included in the article. Further inquiries can be directed to the corresponding author.
